# Olfactory Bulb Volume Reflects Olfactory Dysfunction and Network Organization: Insights From the Population‐Based Rhineland Study

**DOI:** 10.1002/alr.70130

**Published:** 2026-03-02

**Authors:** Weiyi Zeng, Konstantinos Melas, Santiago Estrada, N. Ahmad Aziz, Monique M. B. Breteler

**Affiliations:** ^1^ Population Health Sciences German Center for Neurodegenerative Diseases (DZNE) Bonn Germany; ^2^ AI in Medical Imaging German Center for Neurodegenerative Diseases (DZNE) Bonn Germany; ^3^ Department of Neurology Faculty of Medicine University of Bonn Bonn Germany; ^4^ Institute for Medical Biometry, Informatics and Epidemiology University Hospital Bonn University of Bonn Bonn Germany

**Keywords:** functional connectivity, MRI, odor identification, olfactory bulb, olfactory network

## Abstract

**Background:**

Olfactory dysfunction is common in aging and an early symptom of neurodegenerative diseases, but how structural (olfactory bulb [OB] volume) and functional (olfactory network [OFN] functional connectivity [FC]) brain features interact to shape odor identification ability remains unclear. Therefore, we assessed the interrelations among OB volume, OFN FC, and odor identification ability in a large population‐based cohort.

**Methods:**

Using cross‐sectional data from 5605 participants of the Rhineland Study (age range: 30–95 years), we extracted OB volume and OFN FC from 3T MRI scans. Odor identification was examined with the 12‐item “Sniffin’ Sticks” test. Using linear regression, we examined the relations between OB volume, OFN FC, and odor identification.

**Results:**

A smaller OB was associated with worse odor identification (standardized *β* = 0.09, 95% confidence interval: 0.06–0.12). This association was stronger in men and strongest in older individuals of both sexes. Only in participants with a large OB, lower OFN FC was significantly associated with worse odor identification (standardized *β* between 0.03 and 0.12), especially among older participants (62–95 years) and in memory‐related regions (hippocampus, amygdala, and orbitofrontal cortex).

**Conclusion:**

Our findings demonstrate the importance of OB volume in detecting olfactory dysfunction. Moreover, they reveal that the OB contributes to odor identification both directly and by modulating central network function, offering new insights into olfactory dysfunction as a potential biomarker for neurodegeneration.

## 1 Introduction

Olfactory dysfunction is common in aging, affecting approximately one in four adults [1]. Apart from being a personal burden, it is also a prodromal symptom of age‐associated neurodegenerative disorders [2], and it is associated with cardiovascular diseases [3], frailty [4], and increased mortality [5]. Additionally, as it is a key symptom of coronavirus disease‐2019 (COVID‐19), interest in its pathophysiology and treatment has seen a surge in recent years [6].

Olfaction is a complex process involving not only the peripheral olfactory system but also higher order cortical integration of perceptual and cognitive information [7]. In humans, odor perception is initiated peripherally with the detection of an odorant by the nasal neuroepithelium, with the signals transmitted further to the olfactory bulb (OB) and, subsequently, to the olfactory cortex [8]. Thus, the OB plays a pivotal role in olfactory processing. This is also demonstrated by the consistent association between OB atrophy and olfactory dysfunction, established by multiple studies [9, 10, 11, 12, 13, 14, 15, 16, 17, 18, 19, 20, 21]. Accordingly, we recently reported that OB volume and volumes of different central olfactory structures were associated with odor identification in the general population [21]. The primary olfactory cortex directly receives input from the OB and further projects this input to various cortical regions (e.g., orbitofrontal and insular cortex), which comprise the secondary olfactory cortex [22]. A recent functional magnetic resonance imaging (fMRI) study found strong connections among these regions, which nevertheless remained functionally specialized, exhibiting small‐world characteristics [23]. This small‐world organization ensures efficient information processing and enhances resilience against pathological changes.

Psychophysical evaluations, including odor identification tests, are the most common method for assessing olfactory function in clinical settings because of their validated clinical utility, ease of administration, and availability of normative values for reference [24]. However, their performance is affected by cultural background and requires active cooperation, rendering them less suitable for patients with cognitive impairment. As a result, MRI‐derived OB volume has emerged as a proxy of olfactory performance [8, 25], but its precise role in olfactory processing remains unclear. Recent advances in mapping the olfactory network (OFN) have facilitated exploration of functional connectivity (FC) patterns associated with olfactory function [26, 27]. Yet, how these dynamic network interactions relate to both OB morphology and behavioral performance is also unclear. Better understanding of this relation would deepen insight into the central integration of sensory information. This would aid the development of therapeutic strategies against olfactory dysfunction and improve understanding of its role in neurodegeneration.

Here, we used multimodal neuroimaging to explore how OB volume and central OFN FC contribute to odor identification performance in a large population‐based cohort, accounting for demographic, environmental, and genetic factors. Additionally, we investigated whether and how age and sex modify these relationships to disentangle the heterogeneity of olfactory dysfunction.

## 2 Materials and Methods

### 2.1 Participants

All analyses were performed using baseline data from the Rhineland Study, an ongoing population‐based cohort study in Bonn, Germany. All inhabitants of two geographically defined areas of Bonn, aged 30 years or over, are invited to participate. The only exclusion criterion is insufficient command of the German language to provide informed consent. Approval to undertake the study was obtained from the ethics committee of the University of Bonn, Medical Faculty. The study is carried out in accordance with the recommendations of the International Conference on Harmonization Good Clinical Practice standards. We obtained written informed consent from all participants in accordance with the Declaration of Helsinki.

### 2.2 Analytical Sample

Our study was based on the first 8318 participants of the Rhineland Study, who enrolled between March 2016 and November 2021. After exclusion of participants with missing or incomplete data on the 12‐item “Sniffin’ Sticks” odor identification test (SIT‐12) or MR imaging, our initial analytical sample consisted of 5605 participants (Figure S1).

### 2.3 Image Acquisition

MRI scans from Rhineland Study participants were collected at two examination sites with identical 3T Siemens MAGNETOM Prisma MRI scanners (Siemens Healthcare, Erlangen, Germany) equipped with 64‐channel head–neck coils. For this study, we used as follows: (a) a 3D T1‐weighted (T1w) multi‐echo Magnetization Prepared Rapid Gradient‐Echo sequence; (b) a 3D T2‐weighted (T2w) turbo‐spin‐echo (TSE) sequence; (c) a rapid 3D echo‐planar imaging sequence; and (d) a 3D dual‐echo spoiled GRE sequence (B_0_ field map) [28].

### 2.4 Image Analysis

#### 2.4.1 OB Volume

**FIGURE 1:**
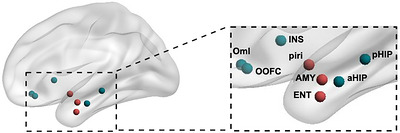
The Olfactory functional network. Three‐dimensional transparent brain model of the right hemisphere (in sagittal view). Zoomed view from the right side shows the anatomical location of the eight ROIs of the olfactory network. The ROIs in the primary olfactory network are marked with red, whereas those in the secondary olfactory network are marked with blue. aHIP, anterior Hippocampus; AMY, amygdala; ENT, entorhinal cortex; INS, insula; Oml, medial lateral orbitofrontal cortex; OOFC, olfactory orbitofrontal cortex; pHIP, posterior Hippocampus; piri, piriform cortex; ROI, region of interest.

OB tissue features were obtained from the predicted segmentation maps using a fully automated post‐processing pipeline based on high‐resolution T2w images and developed by Estrada et al. (Figure 1) [29]. The quality of the output was manually assessed by a single experienced rater (SE), blinded to participants’ olfactory function (SIT‐12 scores), for the entire dataset (Figures S1 and S2). OB volume has been included as the sum of volumes of the left and the right OB. In cases with no apparent OB detection on consecutive slices of an MRI scan, OB volume was defined as 0 (*N* = 16). To adjust for the potential effect of head size, we used the eTIV generated by FreeSurfer 6.0 [30] as a proxy, normalizing the volume of the OB to the study population as follows:

(1) $$\mathrm{OB}\;\mathrm{Volum}e_{\mathrm{adjusted}}=\frac{\mathrm{OB}\;\mathrm{Volume}}{\mathrm{eTIV}}\;\times \;\mathrm{eTI}V_{\mathrm{mean}}$$

#### 2.4.2 Olfactory Network Functional Connectivity

FC measurements were derived from resting‐state fMRI data, which were preprocessed with statistical parametric mapping (SPM12) software (Wellcome Department of Imaging Neuroscience, Institute of Neurology, London). Details about preprocessing and quality control procedures have been previously reported [31].

Eight regions of interest (ROIs) were selected according to Arnold et al. [23], including the primary olfactory areas piriform cortex, amygdala, and entorhinal cortex; and the secondary olfactory regions insula, anterior and posterior hippocampus, olfactory, and medial‐lateral orbitofrontal cortex (Figure 1). For each ROI, the time series were extracted from a 6‐mm radius sphere centered at the centroid coordinates. We then calculated the cross‐correlation for each pair of ROIs’ time series using Pearson's moment correlation, yielding a symmetric 8 × 8 FC matrix, which was subsequently Fisher transformed. All negative correlations were set to zero, and only positive values were retained to avoid spurious anticorrelations [32, 33]. Composite scores, representing FC measures within and between primary and secondary OFNs, were computed as the mean FC measures between seed region pairs in primary and secondary OFNs.

#### 2.4.3 Odor Identification

The SIT‐12 test is a widely used screening test for the assessment of odor identification ability (Figure 1) [34]. In short, 12 felt‐tip sticks from a test kit (Burghart Messtechnik GmbH, Germany), each carrying a distinct odorant, were consecutively positioned in front of both nostrils for 3–4 s by trained technicians in a well‐ventilated room. Participants were then asked to choose only one out of four options for each odorant. The time interval between two consecutive odor presentations was at least 20 s. The final SIT‐12 score was generated as the total number of correct answers (range 0–12).

#### 2.4.4 Demographic, Environmental, and Genetic Information

Age, sex, smoking, alcohol consumption, having an *APOE* ε4 allele, nasal congestion, and recent upper respiratory tract infection (common cold) have all been reported as risk factors for olfactory dysfunction [1, 35, 36, 37]. We, thus, included them as potential confounders when examining the relation between OB volume and olfactory function. Information on smoking status, nasal patency, and recent cold was based on self‐reports. Smoking categories were defined as current smoker, former smoker, and nonsmoker. We determined whether participants without self‐reported smoking status are current smokers by measuring the levels of cotinine in peripheral blood samples. Nasal patency was defined as “blocked” or “free” based on participants’ ratings, with no further information (e.g., unilateral or bilateral blockage) collected. Recent cold was defined as reporting an upper respiratory tract infection in the 6 weeks preceding the SIT‐12 examination. No information was available on participants’ olfactory function prior to the examination. Participants’ alcohol consumption was defined as average amount of pure alcohol, in grams, consumed per day. Participants who reported implausible ranges of daily caloric intake (less than 800 or more than 6000 calories per day) were excluded from analyses including alcohol consumption. *APOE* genotype was determined in blood‐derived DNA samples. We additionally accounted for the confounding effect of first language (German or non‐German) on SIT‐12 examination performance, as the examination was exclusively carried out in German language. To examine previously reported effects of hand preference (handedness) on olfactory function and the OB [38], we analyzed the differences in OB volume, OFN FC, and SIT‐12 score among self‐reported right‐handed, left‐handed, and ambidextrous participants.

#### 2.4.5 Statistical Analysis

To assess the relationships of imaging measures (OB volume, OFN FC measures) with SIT‐12 score and the effect of potential confounders, we fitted multivariable linear regression models separately for each imaging measure and with different sets of covariates: For the base model, we included SIT‐12 score as the dependent variable and imaging measures as the independent variables, adjusting for age, age^2^, sex, nasal patency, and first language; to examine the influence of genetic predisposition, we included *APOE* genotype as a covariate in the base model; to examine the influence of nongenetic covariates, we added variables for alcohol consumption, smoking status, and upper respiratory tract infection to the base model. *APOE* genotype and nongenetic covariates were analyzed in separate models due to the large number of missing or excluded cases, which would lead to a considerably smaller sample and reduced precision when analyzed together. To account for the joint effect of these variables on SIT‐12, we additionally performed a sensitivity analysis in which we included all of them as covariates.

To assess whether the association between imaging measures and SIT‐12 score varied by different age or sex groups, we added interaction terms of age and sex with each imaging measure. To further characterize these potential effects, we performed stratified analyses for age, splitting the sample by age tertiles (young: 30–50 years old, middle: 50–62 years old, old: 62–95 years old), and sex. To examine the associations among very old participants, we also ran a stratified analysis comparing effects between those aged 62–80 and 80–95 years.

Considering OB as the first relay of the olfactory pathway, the size of OB might largely influence the signal reception and transmission to central OFN. Hence, we additionally examined this potential modification effect of OB volume on the association between OFN FC measures and SIT‐12 score by including an interaction term (OB volume × OFN FC). Furthermore, we performed stratified analyses by dichotomizing the cohort into larger and smaller OB volume groups based on median OB volume (53.26 mm^3^).

To account for potential motion effects within the scanner, we included mean framewise displacement (FD) in all models where OFN FC was the primary independent variable.

Because SIT‐12 scores were not normally distributed (Figure S3), the 95% confidence intervals (CI) of all regression coefficients in models where SIT‐12 was the dependent variable were estimated using bias‐corrected accelerated bootstrapping with 6000 resamplings.

We did not apply a correction for multiple testing, as our OFN FC measures were combined in composite scores, minimizing the number of statistical tests performed and thus the need for such a correction. We only tested associations with individual FC measures when the composite scores’ coefficients or their interaction factors were significant.

Prior to analysis, all continuous variables were *z*‐transformed to facilitate the comparison of effect estimates.

All linear regression coefficients are reported as standardized *β* estimates and 95% CI. All statistical analyses were performed in the R programming environment (version 4.3.1).

## 3 Results

The study population characteristics are shown in Table 1. In the sample with complete OB volume and SIT‐12 scores, the mean age was 55.47 years (SD = 13.46 years), ranging from 30 to 95 years, and 3231 (57.6%) of the participants were women. The mean SIT‐12 score was 9.99 (SD = 1.59), whereas mean OB volume was 52.36 mm^3^ (SD = 15.03 mm^3^). Sixteen participants had no apparent OB, whereas the largest OB volume was 118.50 mm^3^. None of the examined variables differed significantly between the overall sample and the subgroup used to evaluate FC.

**TABLE 1:** Study population characteristics.

Variable	Participants with complete OB volume and SIT‐12 data (*n* = 5605)^a^	Participants with complete OB volume, SIT‐12 and FC data (*n* = 5140)^a^	*p* value for intergroup differences^b^
**Age (years), mean (SD)**	55.5 (13.5)	55.2 (13.3)	0.23
**Sex, *n* (%)**			0.23
Men	2374 (42.4)	2118 (41.2)	
Women	3231 (57.6)	3022 (58.8)	
**Nasal patency, *n* ()**			0.85
Blocked	791 (14.1)	719 (14.0)	
Free	4814 (85.9)	4421 (86.0)	
**Recent cold, *n* (%)**	1334 (23.8)	1214 (23.6)	0.82
**SIT‐12, mean (SD) score**	10.0 (1.6)	10.0 (1.6)	0.27
**Smoking status, *n* (%)** ^c^			0.82
Current	692 (12.6)	621 (12.3)	
Former	2163 (39.3)	1972 (39.1)	
Never	2647 (48.1)	2456 (48.6)	
***APOE* genotype, *n* (%)** ^c^			>0.99
Apo‐ε2/ε2	46 (0.9)	43 (0.9)	
Apo‐ε2/ε3	685 (13.3)	620 (13.2)	
Apo‐ε2/ε4	103 (2.0)	96 (2.0)	
Apo‐ε3/ε3	3052 (59.4)	2792 (59.4)	
Apo‐ε3/ε4	1128 (21.9)	1034 (22.0)	
Apo‐ε4/ε4	87 (1.7)	84 (1.8)	
**Alcohol consumption (g/day), mean (SD)** ^c^	19.4 (29.5)	19.1 (29.0)	0.61
**First language, *n* (%)** ^c^			0.73
German	5190 (92.6)	4768 (92.8)	
Other	412 (7.4)	369 (7.2)	
**Handedness, *n* (%)** ^c^			>0.99
Ambidextrous	161 (2.9)	146 (2.9)	
Left	283 (5.2)	261 (5.2)	
Right	5020 (91.9)	4618 (91.9)	
**Left OB volume (mm^3^), mean (SD)**	25.7 (7.9)	25.9 (7.9)	0.26
**Right OB volume (mm^3^), mean (SD)**	26.6 (8.2)	26.8 (8.0)	0.25
**Total OB volume (mm^3^), mean (SD)**	52.3 (15.0)	52.6 (14.9)	0.23

Abbreviations: FC, functional connectivity; OB, olfactory bulb; SIT‐12, 12‐item “Sniffin’ Sticks” odor identification test.

^a^ The sample of participants with complete OB volume, SIT‐12, and FC data is a subset of the sample with complete OB volume and SIT‐12 data.

^b^ Intergroup differences were examined with the Pearson's Chi‐squared test for categorical variables and with the Welch two sample *t*‐test for numeric variables.

^c^ Participants with missing data: smoking status: *n* = 103 (1.8%); *APOE* genotype: *n* = 504 (9.0%); alcohol consumption: *n* = 571 (10.2%); first language: *n* = 3 (0.1%); handedness: *n* = 141 (2.5%).

**FIGURE 2:**
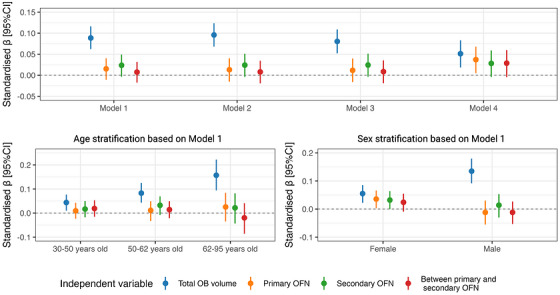
The association of OB volume/olfactory network FC measures with SIT‐12 score, adjusting for covariates. (A) Linear regression coefficients for the association of OB volume/olfactory network FC measures with SIT‐12 score adjusting for demographic covariates (Model 1), APOE (Model 2) status, nongenetic covariates (Model 3), and age and sex interactions (Model 4). (B) Age and (C) sex stratification, performed based on Model 1. The standardized *β* (*x*‐axis) indicates the SD change of SIT‐12 score per SD change of continuous variables, or per level of categorical variables, and the corresponding bootstrapped 95% CI. Model 1: SIT‐12 score ∼ OB volume/FC + age + age^2^ + sex + nasal patency + first language. Model 2: SIT‐12 score ∼ OB volume/FC + age + age^2^ + sex + nasal patency + first language + APOE status. Model 3: SIT‐12 score ∼ OB volume/FC + age + age^2^ + sex + nasal patency + first language + smoking + alcohol consumption + recent upper respiratory tract infection (cold). Model 4: SIT‐12 score ∼ OB volume/FC + age + age^2^ + sex + OB volume/FC × age + OB volume/FC × sex + nasal patency + first language. CI, confidence interval; FC, functional connectivity; OB, olfactory bulb; OFN, olfactory network; SD, standard deviation; SIT‐12, 12‐item “Sniffin’ Sticks” odor identification test.

Larger OB volume was significantly associated with higher SIT‐12 score (*β* = 0.09, 95% CI = 0.06–0.12; Figure 2A and Figure S4). Estimates changed slightly when further adjusted for *APOE* status (*β* = 0.10, 95% CI = 0.07–0.12; Figure 2A and Figure S4), or for alcohol consumption, smoking, upper respiratory tract infection, first language, and nasal patency (*β* = 0.08, 95% CI = 0.05–0.11; Figure 2A and Figure S4). Of note, the *APOE* ε4/ε4 genotype and the *APOE* ε2/ε4 genotype were significantly associated with worse olfactory function. Including all potential confounders in a single model led to very similar results (Figure S4). Contrary to OB volume, none of the OFN FC measures showed significant associations with SIT‐12 score in any of the above models (Figure 2A), except for primary OFN after introducing the interaction term between FC measures with age and sex.

Interestingly, age and sex significantly modulated the association between OB volume and odor identification, with older individuals (62–95 years) and men showing a stronger positive association (Table S1). Although not statistically significant, the association between OFN FC measures and odor identification also appeared to vary by age and sex (Table S1). The stratified analyses showed that the association of larger OB volume with higher SIT‐12 score was progressively stronger with older age groups, with the strongest association observed in the oldest age group (Figure 2B and Table S2). Moreover, the association between larger OB volume and odor identification was nearly threefold stronger in male participants compared to female (Figure 2C and Table S2). In contrast, there were no differences in the association between FC measures and odor identification among different age groups (Figure 2B and Table S2). Compared to male, FC measures within primary OFN and secondary OFN showed stronger associations with SIT‐12 in female participants (Figure 2C and Table S2). When examining very old participants (aged 80–95 years), effects tended to be similar to those aged 62–80 years, but due to wide CIs, no associations reached statistical significance (Figure S5).

**FIGURE 3:**
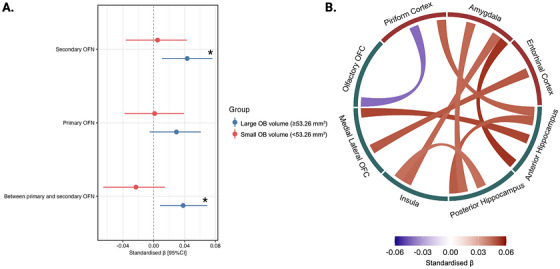
The association between olfactory functional network FC measures and SIT‐12 score. (A) Linear regression coefficients for the association of olfactory network FC measures with SIT‐12 score, stratified by OB volume. The standardized *β* (*x*‐axis) indicates the SD change of SIT‐12 score per SD change of olfactory network FC and the corresponding bootstrapped 95% CI. Variables that reached statistical significance, based on the bootstrapped 95% CI, have been marked with an asterisk (*). (B) Chord diagram for individual FC measures in olfactory network in relation to SIT‐12 score in the participants with larger OB. Colors of the links correspond to standardized beta‐values from the linear regression model: SIT‐12 ∼ FC + age + age^2^ + sex + FD + nasal patency + first language, indicating SD change of SIT‐12 score per SD change in FC measures in the olfactory network. Only FC measures significantly associated with SIT‐12 score are shown. Statistical significance is based on the bootstrapped 95% CI. CI, confidence interval; FC, functional connectivity; OB, olfactory bulb; SD, standard deviation; SIT‐12, 12‐item “Sniffin’ Sticks” odor identification test.

Among all three OFN FC measures, between primary and secondary OFN FC showed a significant interaction with OB volume (*β* = 0.03, 95% CI = 0.01–0.06; Figure S6A). After stratifying the participants in large or small OB volume groups, significant associations between all OFN FC networks and SIT‐12 score were only observed in the participants with larger OB volumes (within secondary OFN: *β* = 0.04, 95% CI = 0.01–0.08; primary‐secondary OFN FC: *β* = 0.04, 95% CI = 0.01 to 0.07; Figure 3A).

We further performed the same OB volume‐stratified analysis separately for the individual FC measures comprising the composite scores. In the larger OB volume group, we identified significant associations with SIT‐12 for several FC measures (Table 2 and Figure 3B).

**TABLE 2:** The association of individual olfactory network functional connectivity with SIT‐12 score.

Functional connectivity	Participants with OB ≥ 53.26 mm^3^ (standardized beta [95% CI])	Participants with smaller OB < 53.26 mm^3^ (standardized beta [95% CI])
ENT_AMY	0.027 [−0.006, 0.057]	−0.004 [−0.04, 0.033]
Insula_AMY	0.041 [0.007, 0.074]	0.01 [−0.03, 0.048]
Insula_ENT	0.023 [−0.009, 0.056]	−0.009 [−0.052, 0.03]
OOFC_AMY	0.028 [−0.005, 0.061]	−0.027 [−0.07, 0.015]
OOFC_ENT	−0.014 [−0.045, 0.018]	−0.025 [−0.068, 0.013]
OOFC_Insula	0.01 [−0.02, 0.039]	0.005 [−0.039, 0.045]
Oml_AMY	0.008 [−0.025, 0.042]	−0.021 [−0.065, 0.022]
Oml_ENT	0.045 [0.013, 0.074]	−0.029 [−0.071, 0.009]
Oml_Insula	0.029 [−0.003, 0.061]	0.024 [−0.015, 0.061]
Oml_OOFC	−0.006 [−0.041, 0.03]	−0.003 [−0.054, 0.04]
aHIP_AMY	0.052 [0.018, 0.085]	0.003 [−0.035, 0.042]
aHIP_ENT	0.012 [−0.021, 0.045]	−0.025 [−0.068, 0.013]
aHIP_Insula	0.006 [−0.024, 0.035]	0.018 [−0.024, 0.059]
aHIP_OOFC	0.002 [−0.031, 0.034]	−0.017 [−0.066, 0.025]
aHIP_Oml	0.047 [0.016, 0.077]	0.013 [−0.026, 0.051]
pHIP_AMY	0.038 [0.003, 0.071]	−0.012 [−0.054, 0.029]
pHIP_ENT	0.025 [−0.006, 0.056]	−0.031 [−0.076, 0.008]
pHIP_Insula	0.039 [0.004, 0.07]	−0.006 [−0.05, 0.037]
pHIP_OOFC	−0.01 [−0.044, 0.024]	−0.013 [−0.056, 0.026]
pHIP_Oml	0.031 [−0.001, 0.061]	−0.015 [−0.064, 0.028]
pHIP_aHIP	0.041 [0.007, 0.073]	0.009 [−0.031, 0.048]
piri_AMY	0.027 [−0.004, 0.059]	−0.018 [−0.061, 0.02]
piri_ENT	0.016 [−0.015, 0.048]	−0.023 [−0.065, 0.015]
piri_Insula	0.012 [−0.02, 0.042]	0.025 [−0.012, 0.062]
piri_OOFC	−0.035 [−0.069, −0.003]	−0.022 [−0.068, 0.02]
piri_Oml	0.013 [−0.023, 0.044]	−0.027 [−0.07, 0.013]
piri_aHIP	0.038 [0.003, 0.069]	−0.012 [−0.061, 0.03]
piri_pHIP	0.019 [−0.015, 0.051]	−0.039 [−0.084, −0.002]

Abbreviations: aHIP, anterior hippocampus; AMY, amygdala; CI, confidence interval; ENT, entorhinal cortex; INS, insula; OB, olfactory bulb; Oml, medial lateral orbitofrontal cortex; OOFC, olfactory orbitofrontal cortex; pHIP, posterior hippocampus; piri, piriform cortex; SIT‐12, 12‐item “Sniffin’ Sticks” odor identification test.

We also explored whether OB volume differentially influenced the association between OFN FC measures and SIT‐12 score across different age or sex strata. The modulatory effect of OB volume on this association was most pronounced in the oldest age group (secondary OFN: *β* = 0.09, 95% CI = 0.03–0.18; primary‐secondary OFN: *β* = 0.12, 95% CI = 0.05–0.20; Figure S6B), whereas no differences were found between female and male participants (Figure S6C).

## 4 Discussion

Harnessing data from a large, population‐based cohort study with a wide age range, we disentangled the relations among OB volume, OFN FC measures, and olfactory function as assessed by odor identification performance. We were able to confirm OB volume as a robust determinant of odor identification ability, independently of age, sex, OFN FC, *APOE* status, and nongenetic factors. Moreover, we found that OFN FC between key limbic regions was related to odor identification, particularly among women and older individuals with large OB. These findings shed new light on the peripheral and central neural circuitry supporting smell recognition and highlight the involvement of memory‐associated regions in successful odor identification.

Our findings confirm that OB size is a key anatomical substrate underlying odor identification ability. The association of smaller OB volume with worse olfactory function agrees with previous findings in smaller cohorts [9, 10, 11, 12, 13, 14, 15, 16, 17, 18, 19, 20, 39, 40, 41, 42], supporting the crucial role of the OB as the first relay in the olfactory pathway regulating olfactory input. Our large sample size enabled more precise effect size estimates. This association reflects the bidirectional relationship between OB volume and olfactory function, as peripheral insults that impair olfactory function can lead to OB atrophy and vice versa [43, 44]. Additionally, it corroborates the potential of OB volume measurements as an objective marker of olfactory function [45], as a prognostic indicator for olfactory recovery [46], or as a predictor of susceptibility to olfactory function loss [47]. Moreover, we confirmed the previously described detrimental effect of older age, male sex, and the *APOE* ε4/ε4 genotype on olfactory function [1, 35, 36, 37]. Notably, we also observed an association of the *APOE* ε2/ε4 genotype, but not ε3/ε4, with worse olfactory function. In contrast with the ε4 allele, the effect of ε2 and ε3 on olfaction is underexplored. However, a previous study on cell cultures found that ε3 promotes neurite outgrowth in the olfactory epithelium to a larger extent than ε2, with ε4 having no effect [48]. Thus, although ε2 is generally regarded as more neuroprotective compared to ε3 [49], this relationship might not be universal. Our results suggest that ε3 is more protective than ε2 for olfaction, negating the detrimental effect of ε4 in heterozygous ε3/ε4 individuals.

Beyond the known direct link between OB volume and odor identification, we also uncovered its modulatory role in central OFN pathway: Larger OB facilitates functional coupling between several key central OFN regions, including the piriform cortex, amygdala, hippocampus, and orbitofrontal cortex, which, in turn, enhances odor identification performance. Moreover, our study highlighted the unique role of the OB beyond the transmission of sensory information to the brain. Changes in FC were related to OB volume not only in regions receiving direct input from OB but also in the higher order olfactory cortex (hippocampus, medial‐lateral orbitofrontal cortex, and insula) that are not directly linked with the OB. The relevance of these regions within the OFN is also supported by our previous study on neuroanatomical basis of olfactory dysfunction in a smaller sample. We found that OB volume is associated with the volumes of the amygdala, hippocampus, insular cortex, and medial orbitofrontal cortex [21]. Thus, the present study demonstrates that the OB is not only structurally linked to these regions but also functionally connected, confirming their importance in the OFN.

Given the established roles of the hippocampus and amygdala in memory and emotion [50, 51, 52], our results point to a model in which olfactory identification performance is not merely a function of primary sensory encoding. Instead, it could be shaped by the integration of sensory and memory processes, conditional upon sufficient structural integrity of the OB. This integration may explain why olfactory deficits often precede cognitive decline in neurodegenerative diseases (e.g., Alzheimer's disease and Parkinson's disease) [53, 54, 55, 56, 57, 58], where early OB degeneration constrains network‐wide communications.

Age emerged as a significant modifier of the relationship between OB volume and odor identification. Moreover, we observed that the modifying effect of OB volume on the association between OFN connectivity and odor identification ability was more pronounced among the oldest participants (62–95 years old). It could be hypothesized that during aging, preserved structural integrity of the OB is not only essential for primary olfactory function but also for enabling the engagement of broader cognitive networks implicated in odor identification. Prior work has emphasized that declines in olfactory cognition with aging are primarily attributed to changes in higher order processing regions, rather than initial sensory detection [59, 60]. Our findings extend this view by proposing that a structurally intact OB may support the effective relay of olfactory information into memory and multisensory systems. Thus, in older adults, reduced OB volume may limit the efficiency of central smell signal processing, suggesting that FC within higher cortical networks may only be able to maintain odor identification when OB integrity is preserved. This hypothesis warrants replication, especially in older population with compromised OB integrity but intact higher order networks.

Furthermore, our results revealed sex‐specific patterns in the structure–function relationship underlying odor identification, with OB volume more strongly associated with SIT‐12 performance in men and OFN FC playing a more pivotal role in women, paralleling accumulating evidence that the olfactory system exhibits sex differences across multiple levels [61, 62]. At the peripheral sensory input stage, evidence in rodents showed that males and females have different patterns of olfactory sensory neuron projections into the OB [63], whereas work in male ferrets demonstrated that males had larger and differently distributed glomeruli within the OB compared to females [64]. At the central level, women showed higher olfactory sensitivity, which was highly correlated with social and emotional well‐being [62, 65]. Furthermore, women have demonstrated greater resilience to olfactory decline in pathological states such as Parkinson's disease [66]. Collectively, these findings could support the hypothesis that men may rely more on the structural integrity of the OB, whereas women may leverage broader cognitive and affective networks. Further investigation on sex‐specific network dynamics is still required.

Our study has both strengths and limitations. As already mentioned, thanks to the implementation of our OB automatic segmentation pipeline, we were able to include a large number of participants and we accounted for a range of potential confounders, increasing the robustness of our findings. Furthermore, our integration of detailed volumetric and functional imaging data enabled a comprehensive evaluation of the central functional representations of olfaction. However, as our data were cross‐sectional, we could not infer causality or assess the central processing of olfactory function over time. Although SIT‐12 is widely used as a reliable and quick screening test for odor identification and is regarded as a valid proxy of olfaction [67], it includes a relatively small number of odorants and may be affected by cultural factors. As a result, it may not efficiently capture all components of olfactory processing [68]. Lastly, our cohort was recruited from two distinct regions in Bonn, Germany. Although this ensured standardized assessment protocols and high‐quality MRI data, it may limit the generalizability of our findings to other populations with different genetic background and environmental exposures. Moreover, as our sample consisted primarily of healthy adults, future studies should investigate more diverse ethnic and geographic populations, as well as patient‐based cohorts.

## 5 Conclusions

Overall, our findings confirm the relevance of OB volume for olfactory function and highlight its pivotal role with OFN connectivity in maintaining smell function. Future interventions targeting olfactory training could be prioritized in individuals with smaller OB volume or disrupted central OFN. Further research assessing the causal directionality of the identified associations and validation in diverse patient populations could pave the way for implementing OB size as a biomarker of olfactory dysfunction and other related neurodegenerative diseases in clinical practice.

## Funding

The Rhineland Study is funded by the German Center for Neurodegenerative Diseases (DZNE). This study was further supported by the Federal Ministry of Education and Research grant (grant number: 01KX2230) with the title “PreBeDem—Mit Prävention und Behandlung gegen Demenz” and the Helmholtz Association under the 2023 InnovationsPool.

## Conflicts of Interest

The authors declare no conflicts of interest.

## Supporting information




**Supplementary Figure S1**: Selection of study population. The initial cohort consisted of the first 8318 participants of the Rhineland Study. Abbreviations: SIT‐12, 12‐item “Sniffin' Sticks” odor identification test; MRI, Magnetic Resonance Imaging; eTIV, estimated Total Intracranial Volume, fMRI: functional Magnetic Resonance Imaging. **Supplementary Figure S2**: Visual inspection screenshot of automated OB segmentation. Panel A exhibits the segmentation from sagittal, coronal and axial view around the centroid of predicted segmentation. **Supplementary Figure S3**: Distribution of SIT‐12 score in female (n=3,231, Panel A) and male (n=2374, Panel B) participants. **Supplementary Figure S4**: The association of OB volume with olfactory function, adjusting for covariates separately and in a single model. **Supplementary Figure S5**: The association of OB volume and olfactory network functional connectivity with SIT‐12 score, between age group 62 to 80 years (62–80) and age group 80 to 95 years(80+). **Supplementary Figure S6**: The association of OB volume and olfactory network functional connectivity with olfactory function (Panel A), with the interaction term stratified by age (Panel B) and sex (Panel C). **Table S1**: The association of OB volume/olfactory network functional connectivity with SIT‐12 score. **Table S2**: The association of OB volume/olfactory network functional connectivity with SIT‐12 score across age/sex groups.1
