# Supplementary material for: Olfactory Bulb Volume Reflects Olfactory Dysfunction and Network Organization: Insights From the Population‐Based Rhineland Study
Source: Int Forum Allergy Rhinol. 2026 Mar 2;16(8):809–19. doi: 10.1002/alr.70130 (PMC13432549; doi:10.1002/alr.70130)
Supplement: Supplementary file 1 — Supplementary Figure S1: Selection of study population. The initial cohort consisted of the first 8318 participants of the Rhineland Study. Abbreviations: SIT‐12, 12‐item “Sniffin' Sticks” odor identification test; MRI, Magnetic Resonance Imaging; eTIV, estimated Total Intracranial Volume, fMRI: functional Magnetic Resonance Imaging. Supplementary Figure S2: Visual inspection screenshot of automated OB segmentation. Panel A exhibits the segmentation from sagittal, coronal and axial view around the centroid of predicted segmentation. Supplementary Figure S3: Distribution of SIT‐12 score in female (n=3,231, Panel A) and male (n=2374, Panel B) participants. Supplementary Figure S4: The association of OB volume with olfactory function, adjusting for covariates separately and in a single model. Supplementary Figure S5: The association of OB volume and olfactory network functional connectivity with SIT‐12 score, between age group 62 to 80 years (62–80) and age group 80 to 95 years(80+). Supplementary Figure S6: The association of OB volume and olfactory network functional connectivity with olfactory function (Panel A), with the interaction term stratified by age (Panel B) and sex (Panel C). Table S1: The association of OB volume/olfactory network functional connectivity with SIT‐12 score. Table S2: The association of OB volume/olfactory network functional connectivity with SIT‐12 score across age/sex groups.1 [file ALR-16-809-s001.docx]

**Olfactory bulb volume reflects olfactory dysfunction and network organization: Insights from the population-based Rhineland Study**

Weiyi Zeng, MSc^a,1^, Konstantinos Melas, MSc^a,1^, Santiago Estrada, PhD ^a,b^, N. Ahmad Aziz, MD, PhD^a,c^, and Monique M.B. Breteler, MD, PhD^a,d*^

^1^These authors contributed equally to this work.

^a^ Population Health Sciences, German Centre for Neurodegenerative Diseases (DZNE), Bonn, Germany

^b^ AI in Medical Imaging, German Center for Neurodegenerative Diseases (DZNE), Bonn, Germany

^c^ Department of Neurology, Faculty of Medicine, University of Bonn, Bonn, Germany

^d^ Institute for Medical Biometry, Informatics and Epidemiology (IMBIE), Faculty of Medicine, University of Bonn, Bonn, Germany

**Supplementary Materials**

Supplementary Figure S1-S6

Supplementary Table S1-S2


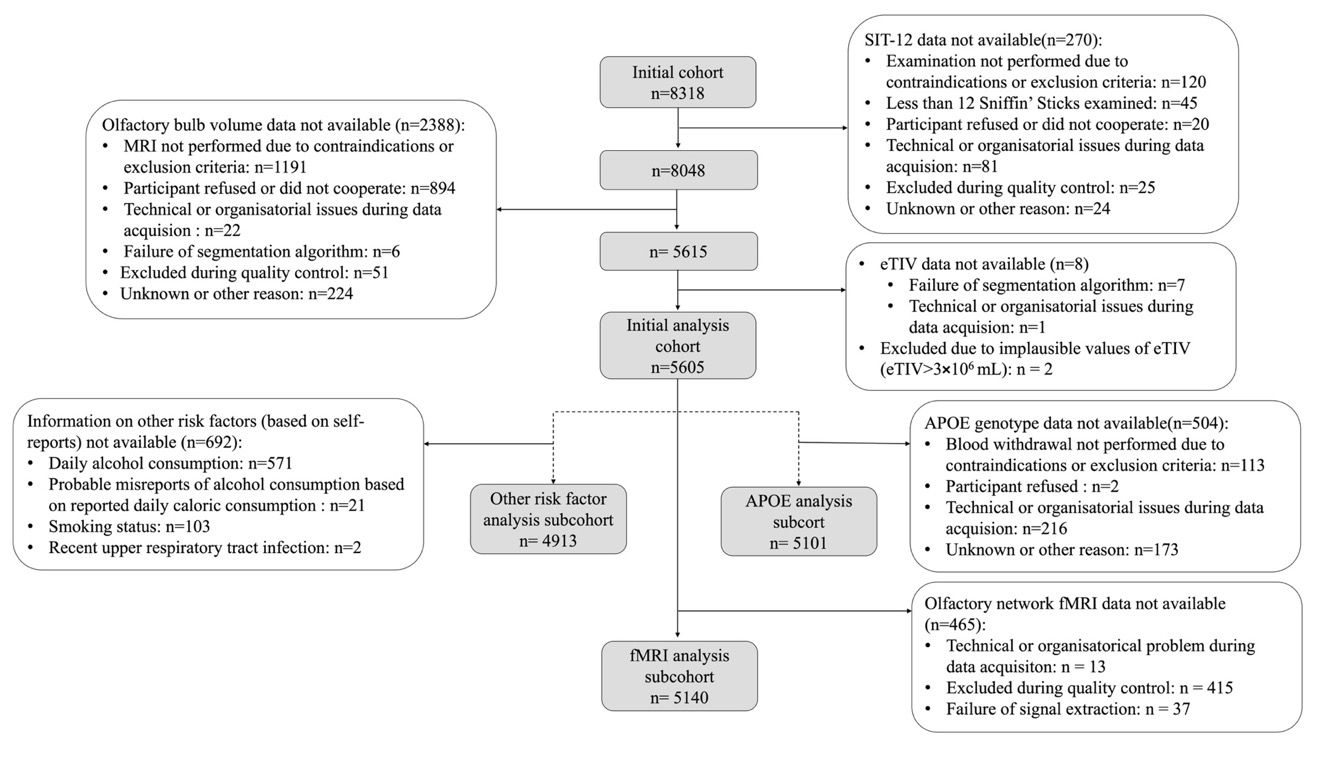


Supplementary Figure S1. Selection of study population. The initial cohort consisted of the first 8318 participants of the Rhineland Study.

Abbreviations: SIT-12, 12-item “Sniffin’ Sticks” odor identification test; MRI, Magnetic Resonance Imaging; eTIV, estimated Total Intracranial Volume, fMRI: functional Magnetic Resonance Imaging


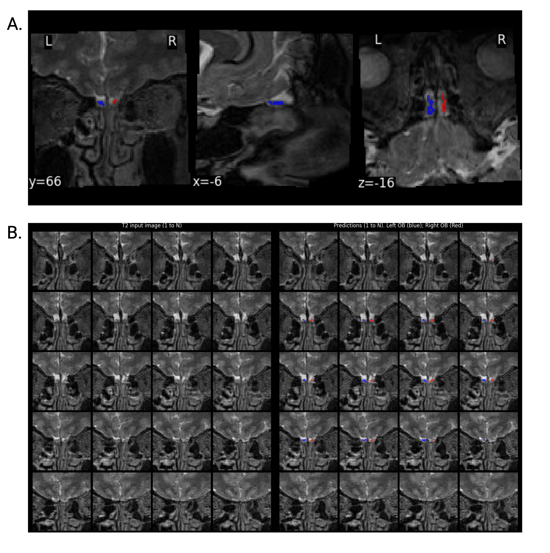


Supplementary Figure S2. Visual inspection screenshot of automated OB segmentation. Panel A exhibits the segmentation from sagittal, coronal and axial view around the centroid of predicted segmentation. Panel B exhibits all coronal MRI slices containing OB tissue. (blue: left OB; red: right OB)

Abbreviations: OB: Olfactory Bulb.


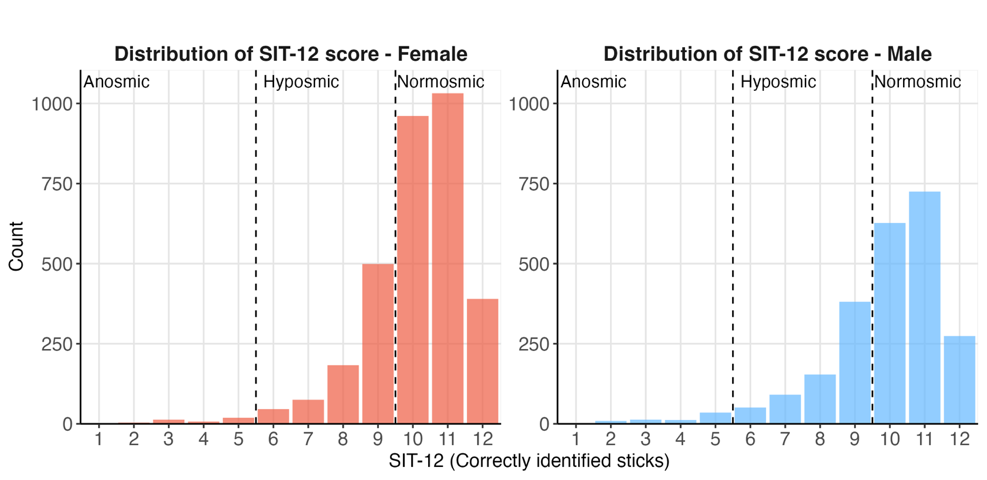


B.

A.

Supplementary Figure S3. Distribution of SIT-12 score in female (n=3,231, Panel A) and male (n=2374, Panel B) participants.

Abbreviations: SIT-12, 12-item “Sniffin’ Sticks” odor identification test


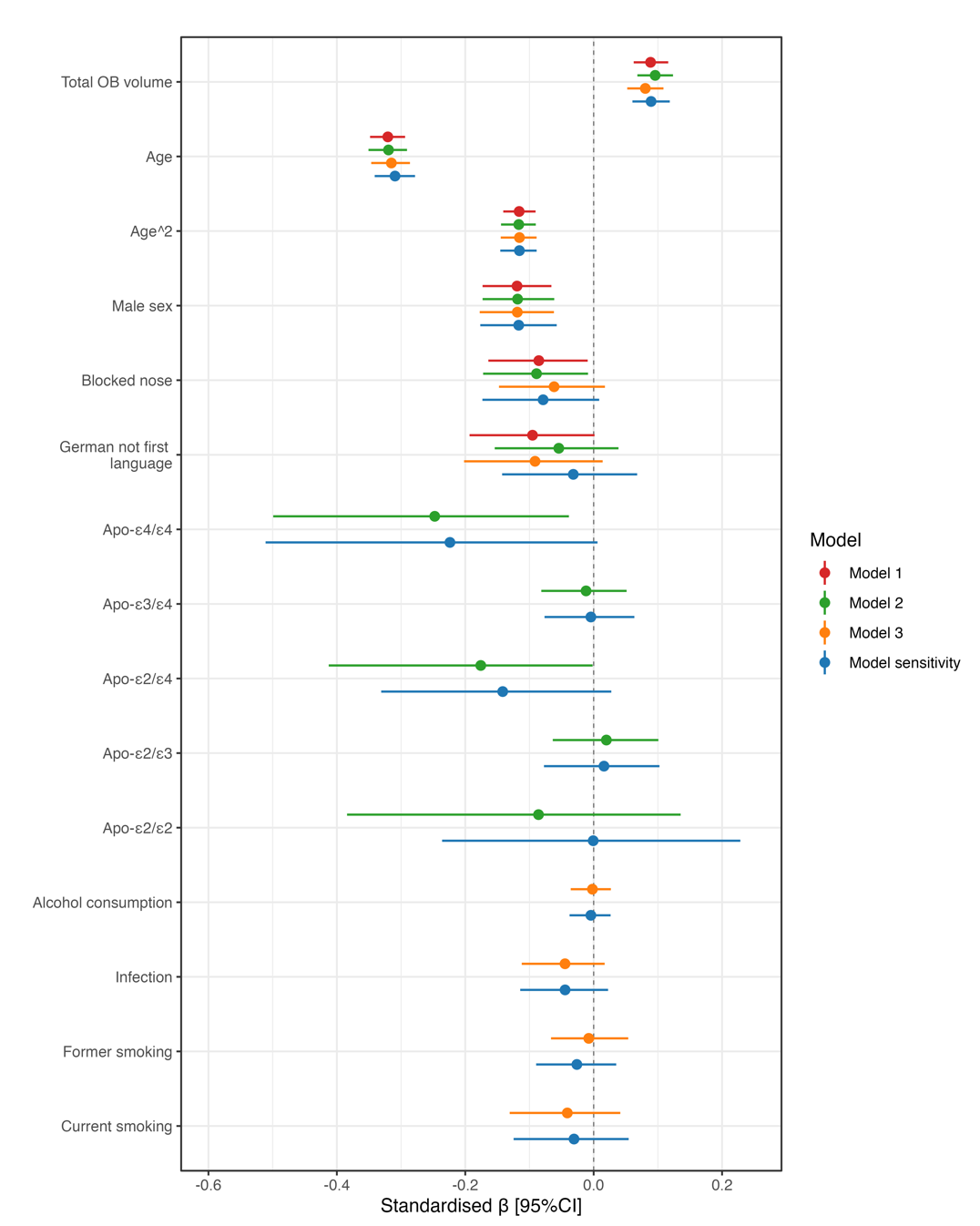


Supplementary Figure S4: The association of OB volume with olfactory function, adjusting for covariates separately and in a single model. Linear regression coefficients for the association of OB volume with SIT-12 adjusting for age, age2, sex, nasal patency and first language (Model1), APOE status (Model 2), non-genetic covariates (Model 3), and APOE status combined with non-genetic covariates in a single model (Model sensitivity). The standardized β (x-axis) indicates the SD change of SIT-12 score per SD change of continuous variables, or per level of categorical variables, and the corresponding bootstrapped 95% CI. Variables that reached statistical significance, based on the bootstrapped 95% CI.

Model 1: SIT-12 score ~ OB volume + age + age^2^ + sex + nasal patency + first language

Model 2: SIT-12 score ~ OB volume + age + age^2^ + sex + nasal patency + first language + APOE status

Model 3: SIT-12 score ~ OB volume + age + age^2^ + sex + nasal patency + first language + smoking + alcohol consumption + recent upper respiratory tract infection (cold)

Model Sensitivity: SIT-12 score ~ OB volume + age + age^2^ + sex + first language + APOE status + nasal patency + smoking + alcohol consumption + recent upper respiratory tract infection (cold)

Abbreviations: SD, Standard Deviation; OB, Olfactory Bulb; SIT-12, 12-item “Sniffin’ Sticks” odor identification test; CI, Confidence Interval


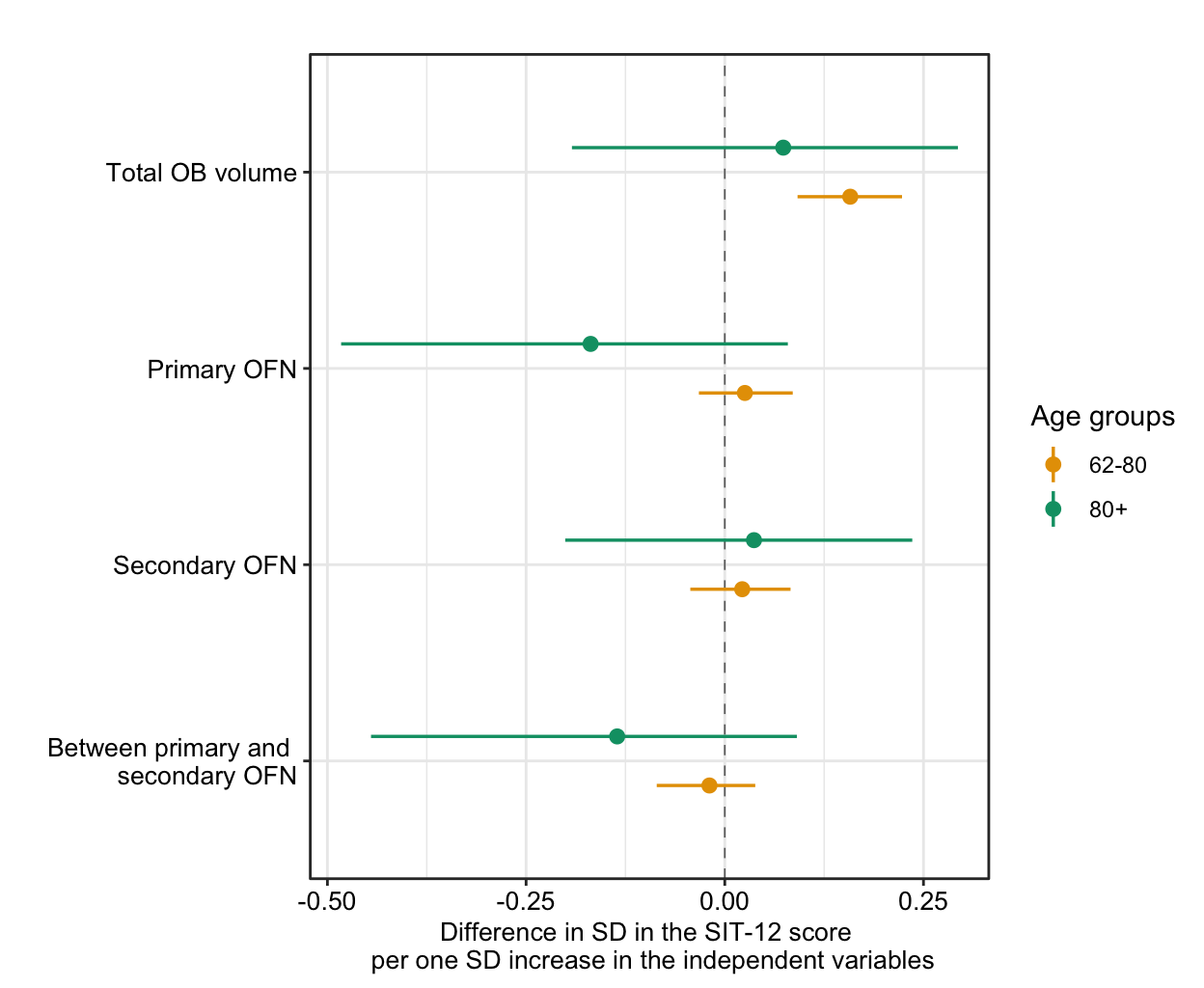


**Supplementary Figure S5: The association of OB volume and olfactory network functional connectivity with SIT-12 score, between age group 62 to 80 years (62-80) and age group 80 to 95 years (80+).** Linear regression coefficients for the association of OB volume/olfactory network FC measures with SIT-12 score adjusting for demographic covariates (Model: SIT-12 score ~ OB volume/FC + age + age^2^ + sex + nasal patency + first language). The standardized β (x-axis) indicates the SD change of SIT-12 score per SD change of continuous variables, and the corresponding bootstrapped 95% CI.

Abbreviations: OB, Olfactory bulb; SIT-12, 12-item “Sniffin’ Sticks” odor identification test; FC, Functional connectivity; SD, Standard deviation; CI, Confidence interval; OFN, Olfactory Network.


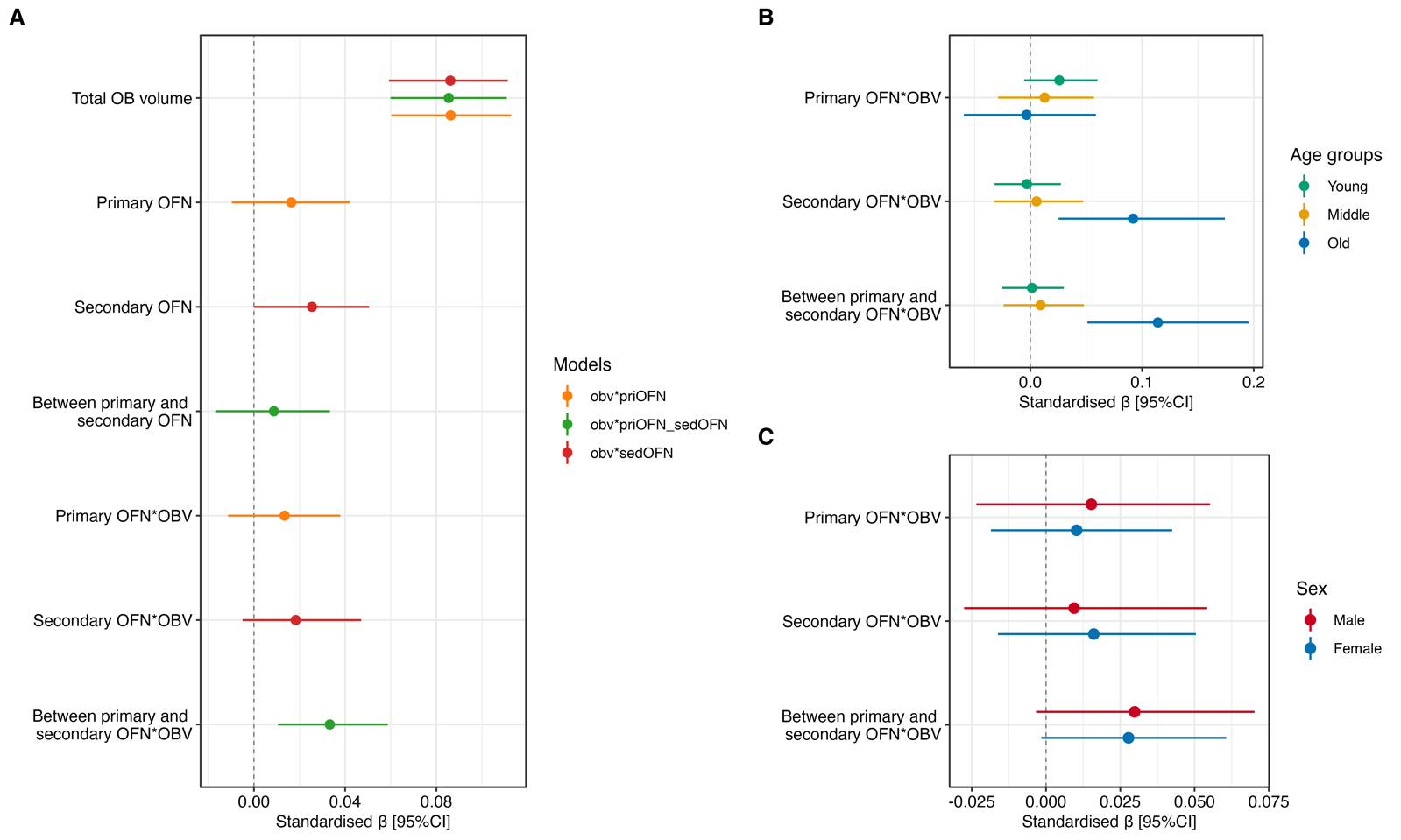


**Supplementary Figure S6:** **The association of OB volume and olfactory network functional connectivity with olfactory function (Panel A), with the interaction term stratified by age (Panel B) and sex (Panel C).** (A) Linear regression coefficients for the association of OB volume and olfactory network functional connectivity including an interaction term with SIT-12 adjusting for age, age^2^, sex, nasal patency and first language. (B) Linear regression coefficients for the interaction between OB volume and olfactory network FC in three different age tertiles (young: 30-50, middle: 50-62, old: 62-90). (C) Linear regression coefficients for the interaction between OB volume and olfactory network FC in male and female groups. The standardized β (x-axis) indicates the SD change of SIT-12 score per SD change of continuous variables, or per level of categorical variables, and the corresponding bootstrapped 95% CI. Variables that reached statistical significance, based on the bootstrapped 95% CI. Model: SIT-12 ~ OB volume + OFN FC + OB volumeOFN FC + age + age^2^ + sex + nasal patency + first language + FD

Abbreviations: SD, Standard Deviation; OB, Olfactory Bulb; SIT-12, 12-item “Sniffin’ Sticks” odor identification test; CI, Confidence Interval, OFN, Olfactory network; FC, functional connectivity; FD, framewise displacement

**Table S1.** **The association of OB volume/olfactory network functional connectivity with SIT-12 score.**

|  | **Adjusted for age, age2, sex, nasal patency, first language (Base model)**  **(beta [95% CI])** | **Base model, additionally adjusted for APOE**  **(beta [95% CI])** | **Base model additionally adjusted for smoking status, alcohol consumption, common cold (beta [95% CI])** | **Interaction term imaging measure * male sex**  **(beta [95% CI])** | **Interaction term imaging measure * age**  **(beta [95% CI])** |
| --- | --- | --- | --- | --- | --- |
| **OB volume** | 0.089 [0.062, 0.116] | 0.096 [0.068, 0.124] | 0.080 [0.052, 0.109] | 0.099 [0.047, 0.155] | 0.046 [0.016, 0.075] |
| **Primary OFN FC** | 0.015 [-0.011, 0.040] | 0.013 [-0.015, 0.040] | 0.012 [-0.016, 0.040] | -0.050 [-0.101, 0.001] | 0.004 [-0.027, 0.032] |
| **Secondary OFN FC** | 0.024 [-0.004, 0.049] | 0.024 [-0.004, 0.051] | 0.024 [-0.004, 0.051] | -0.010 [-0.063, 0.041] | -0.005 [-0.034, 0.025] |
| **Between primary and secondary OFN FC** | 0.008 [-0.017, 0.032] | 0.008 [-0.019, 0.034] | 0.009 [-0.019, 0.035] | -0.047 [-0.100, 0.004] | -0.022 [-0.051, 0.006] |

**Table S2.** **The association of OB volume/olfactory network functional connectivity with SIT-12 score across age/sex groups.^1^**

| **Age stratified** | |
| --- | --- |
| **OB volume** |  |
| 30-50 years | 0.044 [0.010, 0.077] |
| 50-62 years | 0.083 [0.043, 0.125] |
| 62-95 years | 0.157 [0.094, 0.222] |
| **Primary OFN FC** |  |
| 30-50 years | 0.010 [-0.023, 0.043] |
| 50-62 years | 0.011 [-0.033, 0.049] |
| 62-95 years | 0.026 [-0.035, 0.084] |
| **Secondary OFN FC** |  |
| 30-50 years | 0.017 [-0.017, 0.050] |
| 50-62 years | 0.032 [-0.008, 0.069] |
| 62-95 years | 0.022 [-0.043, 0.082] |
| **Between primary and secondary OFN FC** |  |
| 30-50 years | 0.019 [-0.015, 0.053] |
| 50-62 years | 0.014 [-0.021, 0.049] |
| 62-95 years | -0.020 [-0.086, 0.041] |
| **Sex stratified** | |
| **OB volume** |  |
| Female | 0.055 [0.022, 0.085] |
| Male | 0.135 [0.092, 0.180] |
| **Primary OFN FC** |  |
| Female | 0.036 [0.003, 0.066] |
| Male | -0.012 [-0.055, 0.030] |
| **Secondary OFN FC** |  |
| Female | 0.032 [0.000, 0.064] |
| Male | 0.014 [-0.030, 0.053] |
| **Between primary and secondary OFN FC** |  |
| Female | 0.024 [-0.009, 0.055] |
| Male | -0.012 [-0.053, 0.027] |

^1^ Linear regression results are shown as β [95% Confidence Interval]. Models have been adjusted for age, age^2^, sex (if applicable), nasal patency, first language, and framewise displacement (if applicable).

Abbreviations: OB, Olfactory Bulb; SIT-12, 12-item “Sniffin’ Sticks” odor identification test;

OFN, Olfactory Network; FC, Functional Connectivity
